# Impact of Mitophagy and Mitochondrial Unfolded Protein Response as New Adaptive Mechanisms Underlying Old Pathologies: Sarcopenia and Non-Alcoholic Fatty Liver Disease

**DOI:** 10.3390/ijms21207704

**Published:** 2020-10-18

**Authors:** Rodrigo Urbina-Varela, Nataly Castillo, Luis A. Videla, Andrea del Campo

**Affiliations:** 1 Laboratorio de Fisiología y Bioenergética Celular, Departamento de Farmacia, Facultad de Química y de Farmacia, Pontificia Universidad Católica de Chile, Santiago 7810000, Chile; rodrigo.urbina.83@gmail.com (R.U.-V.); nvcastillo@uc.cl (N.C.); 2 Molecular and Clinical Pharmacology Program, Institute of Biomedical Sciences, Faculty of Medicine, University of Chile, Santiago 8380453, Chile; lvidela1944@gmail.com

**Keywords:** mitochondrial homeostasis, aging, liver disease, skeletal muscle

## Abstract

Mitochondria are the first-line defense of the cell in the presence of stressing processes that can induce mitochondrial dysfunction. Under these conditions, the activation of two axes is accomplished, namely, (i) the mitochondrial unfolded protein response (UPR^mt^) to promote cell recovery and survival of the mitochondrial network; (ii) the mitophagy process to eliminate altered or dysfunctional mitochondria. For these purposes, the former response induces the expression of chaperones, proteases, antioxidant components and protein import and assembly factors, whereas the latter is signaled through the activation of the PINK1/Parkin and BNIP3/NIX pathways. These adaptive mechanisms may be compromised during aging, leading to the development of several pathologies including sarcopenia, defined as the loss of skeletal muscle mass and performance; and non-alcoholic fatty liver disease (NAFLD). These age-associated diseases are characterized by the progressive loss of organ function due to the accumulation of reactive oxygen species (ROS)-induced damage to biomolecules, since the ability to counteract the continuous and large generation of ROS becomes increasingly inefficient with aging, resulting in mitochondrial dysfunction as a central pathogenic mechanism. Nevertheless, the role of the integrated stress response (ISR) involving UPR^mt^ and mitophagy in the development and progression of these illnesses is still a matter of debate, considering that some studies indicate that the prolonged exposure to low levels of stress may trigger these mechanisms to maintain mitohormesis, whereas others sustain that chronic activation of them could lead to cell death. In this review, we discuss the available research that contributes to unveil the role of the mitochondrial UPR in the development of sarcopenia, in an attempt to describe changes prior to the manifestation of severe symptoms; and in NAFLD, in order to prevent or reverse fat accumulation and its progression by means of suitable protocols to be addressed in future studies.

## 1. Introduction

Highly metabolic tissues concentrate their energy demands on the participation of mitochondria, obtaining the majority of ATP through the electron transport chain coupled to ATP synthase, pointing to mitochondrial homeostasis as a key phenomenon to sustain organ functions. Imaging studies in living cells showed that mitochondria are in constant motion and undergo structural changes, depending on the cellular energy state [1]. The mitochondrial network moves to places with higher energy requirements along the cytoskeleton, allowing individual mitochondrion to merge with the mitochondrial network on both its external and internal membranes during this trafficking [2]. Evidence shows that the modification of the cellular metabolic state somehow determines the structure of the mitochondrial network, suggesting that each metabolic event could be associated with its own mitochondrial morphology [3]. This situation is clearly illustrated by the observation where the morphology of the mitochondrial network of various cell types rich in mitochondria depends on the energy demands to which these tissues are exposed. Cells in tissues with a high energy demand such as cardiomyocytes and myocytes exhibit a fused mitochondrial morphology, where there are a few very elongated tubules that group almost all of the mitochondria. Instead, cells in tissues that mainly metabolize digested nutrients from the diet, such as hepatocytes, have a fissioned mitochondrial network, where numerous and small isolated mitochondria coexist [4].

Mitochondrial biogenesis is the generation of new mitochondria. It is a critical process for development and differentiation, as well as being a response to eventual mitochondrial dysfunction as it restricts the transmission of harmful mtDNA mutations. Thus, an altered and dysfunctional mitochondrial biogenesis can cause severe disorders, including different pathologies, such as neuromuscular diseases [5]. Regulatory factors of mitochondrial biogenesis are the transcriptional family of the peroxisome proliferator-activated receptor γ (PPARγ) coactivator alpha (PGC-1α), which interacts with NRF-1 and NRF-2 to regulate protein expression. So, the generation of new mitochondria is a coordinated control of several pathways, where PGC-1α improves transcription by facilitating the interaction with the nuclear receptor family through specific LXXLL recognition domains [6]. Mitochondria form an independent compartment in the cell, providing its own translation and protein quality control machinery, including chaperones and proteases inside the mitochondrial matrix, which maintain mitochondrial homeostasis (proteostasis) [7]. In this respect, maintaining optimal protein structure for its adequate function in all cellular compartments including mitochondria is an absolute requisite for cell viability. Different types of chemical modifications occur in cellular proteins, including reversible ones, such as phosphorylation/dephosphorylation reactions involved in metabolic regulation. However, irreversible alterations are usually associated with loss of protein function and cell viability. One of them is the oxidation of target amino acids, which leads to partial protein unfolding or misfolding [8]. These alterations are due to the loss of the secondary and tertiary structures in the oxidative attack domains, which can be assessed by detecting individual modified amino acids [8] or by the spectrophotometric determination of protein carbonyls [9]. Enhancement in cellular protein oxidation in pro-oxidant conditions such as oxidative stress [10] implies proteome instability with loss of function, representing a stress condition at subcellular sites such as endoplasmic reticulum (ER) [11] and mitochondria [12]. Under these circumstances, cellular sensors are activated to trigger a transcriptional response known as unfolded protein response at ER (UPR^er^) and mitochondrial (UPR^mt^) sites, which promote cell survival through the recovery of organelle networks for optimal cellular function [11,12,13,14].

Proteostasis requires several molecular mechanisms to control protein folding, including the adaptation of protein synthesis and degradation, and processes facilitating the post-translational folding and maturation of proteins [7,12,13,14]. The UPR^mt^ is rapidly activated in a time-course of hours to achieve proteostasis, thus representing an acute response that is executed through a multi-axes program [15], which induces pro-death signals under conditions of proteostasis failure or prolonged UPR activation [7,16]. Besides the excessive production of reactive oxygen species (ROS) leading to a proteotoxic stress, UPR^mt^ can also be induced by depletion of oxidative phosphorylation (OXPHOS) components and loss of mitochondrial DNA (mtDNA). Recent evidence has shown that the decline in mitophagy because of the aging process can contribute as a cause of mitochondrial proteostasis [17]. In this regard, the accumulation of dysfunctional mitochondria could be due to impairment in mitophagy flux because of a decrease in mitophagy related proteins [17]. The precise mechanism through which mitophagy is linked to UPR^mt^ is not yet unveiled. Some studies have shown that the inhibition of mitochondrial protease HSP90 induces PINK1 accumulation, ubiquitin phosphorylation at Ser65, Parkin activation and its recruitment to mitochondria, promoting mitophagy [18]. In this way, the inhibition of UPR^mt^ and the subsequent loss of mitochondrial proteostasis, might produce dysfunctional mitochondria that can be a target for ubiquitination and mitophagy. Thus, if the mitochondrion is beyond repair, the entire organelle must be removed before it becomes cytotoxic and causes cellular damage [19]. In the aging process, mitophagy is significantly decreased, causing the accumulation of dysfunctional mitochondria which in turn has defective proteostasis and promotes the activation of the UPR^mt^. The excessive activation of this mechanism may also be detrimental to the cell [20]. In these scenarios, deleterious responses that underlie many pathological states, including sarcopenia and non-alcoholic fatty liver disease (NAFLD), can be activated (Table 1). These age-associated diseases are also characterized by the progressive loss of organ function due to the accumulation of ROS-induced damage to biomolecules, since the ability to counteract the continuous and large generation of ROS becomes increasingly inefficient with aging, positioning mitochondrial dysfunction as a central pathogenic mechanism for both pathological conditions.

### 1.1. Mitochondrial Proteostasis

Mitochondria have been extensively described as the powerhouse of the cell, and mitochondrial function has been placed as a reflection of the cell status [31]. In this regard, mitochondria are in charge of ATP production for muscle contraction, the maintenance of metabolic homeostasis, proper cellular functioning and viability [32]. Furthermore, mitochondria are the first-line responders to different stresses [33] and can adjust bioenergetics, thermogenesis, and oxidative stress responses in order to regulate perturbed homeostasis [34]. Thus, the mitochondrial network has developed mechanisms to combat impending mitochondrial dysfunction [35]. Since the mitochondrial genome encodes 13 proteins that are components of the OXPHOS respiratory complexes, the remaining mitochondrial proteome is encoded by nuclear genes, synthesized in the cytosol and imported into the different mitochondrial compartments through translocase of the outer membrane (TOM) and translocase of the inner membrane (TIM) translocators. Therefore, since most of the proteins in the mitochondria are synthesized by the cytosolic ribosomes, an adequate translocation of proteins to the mitochondria is essential. Mitochondrial biogenesis requires the import and trafficking of proteins synthesized in the cytosol into the intramitochondrial compartments. Proteins destined to the mitochondrial matrix are targeted to the inner membrane Tim17/23 translocon by their pre-sequences [36]. This is carried out using a mitochondrial targeting sequence (MTS), which is located at the N-terminal position of the protein to be imported. This positively charged 25–35 amphipathic amino acid sequence interacts with the TOM complex prior to import. Then, by going through the TOM complex in an unfolded state, the precursor proteins are translocated through the TIM23 complex, which requires the inner mitochondrial membrane (IMM) potential generated by the respiratory chain. The precursor proteins are then translocated to the mitochondrial matrix requiring a chaperone located in it, called mtHsp70, which interacts directly with the TIM23 complex [37,38]. Recent studies have also described the participation of TIM50 in the regulation of the translocation into mitochondria [39], details about translocation of proteins into mitochondria through TIM23 complex in detail can be found in reference [38]. Once in the matrix, the MTS is cleaved by the mitochondrial processing peptidase (MPP) [16]. Thus, chaperones and proteases play fundamental roles in protein folding after import. In this context, the import of most of the mitochondrial intermembrane space (IMS) proteins is mediated by the mitochondrial disulfide relay system linked to oxidative processes [40]. Moreover, mitochondria possess their own protein translation and quality control machinery depending on chaperones and proteases which degrade damaged or misfolded proteins, such as the AAA, LON and ClpXP proteases. These proteins located within the mitochondrial matrix are responsible for maintaining an adequate mitochondrial proteostasis [41].

### 1.2. Mitochondrial Unfolded Protein Response Signaling

**Figure 1:**
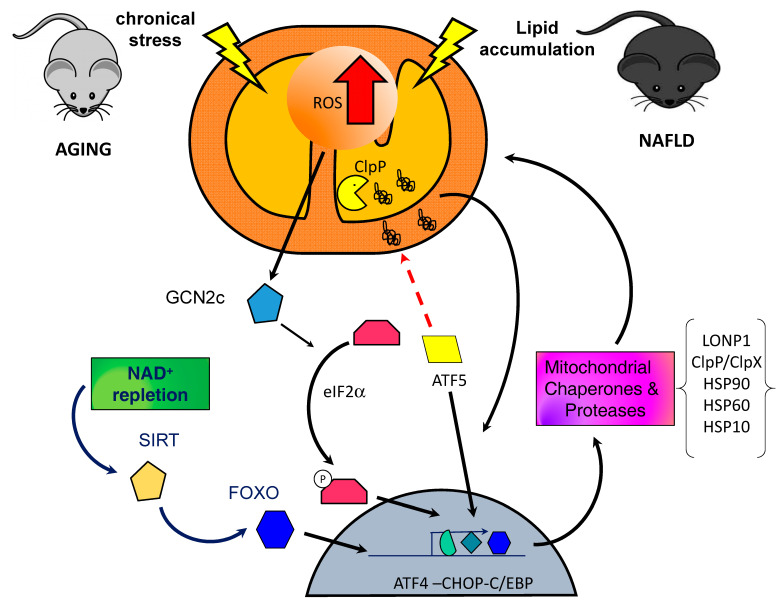
Proposed model of mitochondrial unfolded protein response (UPR^mt^) increased activation in non-alcoholic fatty liver disease (NAFLD) and Sarcopenia. The mechanisms that regulate the activation of UPR^mt^ are still unveiled and are probably determined by the stress conditions present in the cell environment in a context-dependent way. Lipid accumulation in the liver and the presence of chronical stress during the aging process in skeletal muscle cells promote an increase in mitochondrial reactive oxygen species (ROS) production which triggers the phosphorylation of eukaryotic translation initiation factor 2 alpha (eIF2α) to trigger the integrated stress response (ISR). Moreover, the accumulation of misfolded and oxidized proteins which cannot be degraded inside the mitochondria by mitochondrial proteases promotes the translocation of activating transcription factor 5 (ATF-5) to the nucleus, increasing the transcription of mitochondrial chaperones and proteases. The prolonged exposure to the stress may continuously stimulate the response enhancing its effect until making it detrimental for the cell.

The transcriptional regulation of the UPR^mt^ in mammalian cells is comparable to that of Caenorhabditis elegans and, likewise, is mediated by multiple bZIP transcription factors [27]. A functional ortholog of ATFS-1, activating transcription factor 5 (ATF-5), was discovered in mammals. It regulates mitochondrial import efficiency similar to what ATFS-1 does. Under non-stressful conditions, ATF-5 is actively imported into mitochondria for further degradation. However, in the presence of stress signals, it migrates to the nucleus triggering the transcription of various factors associated with mitochondrial proteostasis [27]. Among the transcription factors that have been linked to the UPR^mt^, ATF-5 stands out as the main signaling agent. However, also other participants can be listed as UPR^mt^ responders: (i) CEBP homologous protein (CHOP), (ii) CCAAT/enhancer binding protein β (CEBPβ), (iii) and activating transcription factor 4 (ATF4) [30]. The expression of CHOP and ATF4 has also been linked to a process called integrated stress response (ISR), which is a mechanism dependent on phosphorylation of the eukaryotic translation initiation factor 2 alpha (eIF2α). In this regard, there is evidence that the ISR is necessary for the activation of UPR^mt^ in response to mitochondrial dysfunction in mammals [42,43]. The activation of the ISR is mediated by four kinases that phosphorylate eIF2α in response to specific alterations. Particularly, the ISR kinase, protein kinase RNA-like ER kinase (PERK), responds to the accumulation of misfolded proteins in the ER; protein kinase RNA-activated (PKR) responds to cytosolic double stranded RNA; heme-regulated inhibitor kinase (HRI) is activated by depletion of the heme group [42], and general control non-derepressible 2 kinase (GCN2) is activated by mitochondrial stress, amino acid depletion, ROS and ribosome stalling [44,45]. Following eIF2α phosphorylation by one of these kinases, global protein synthesis is diminished, but the preferential translation of alternative reading frames coding for the transcription factors CHOP, ATF-4 and ATF-5 is preserved, which are associated with the induction of genes related to the UPR^mt^ [26,43,46,47]. Furthermore, UPR^mt^ produces a significant increase in various mitochondrial proteases including lon protease 1 (LONP1) [48] and ClpP/ClpXP; and chaperones heat shock protein 90 (Hsp90), mitochondrial heat shock protein 70 (mtHsp70), Hsp60 and Hsp10 [49] (Figure 1). The UPR^mt^ has been described as a beneficial signaling pathway and it has also been linked to improvement in lifespan and in metabolic homeostasis. Nevertheless, a chronic increase in the UPR^mt^ due to the constant presence of a stressor has been suggested to be detrimental to the cell [20,28].

Mitochondria continually generate reactive oxygen species (ROS), which could eventually be detrimental for proteins, lipids, and DNA. Considering that damaged electron transport chain (ETC) proteins can produce even more ROS, mitochondria use quality control proteases to remove these potentially harmful proteins and respond to the protein stress deployed in the mitochondrial matrix [50]. Additionally, a mito-cellular response to cope with damaged outer mitochondrial membrane (OMM) proteins is carried out by the ubiquitin–proteasome quality control pathway [51], while some mitochondria also respond to genotoxic damage through DNA-repairing pathways found in the nucleus, recovering the function of the organelles [50]. Finally, autophagy is considered as the most radical quality control mechanism, signifying the total elimination of dysfunctional mitochondria (mitophagy).

### 1.3. Mitophagy

Mitophagy is the selective autophagic degradation of mitochondria and mediates the elimination of dysfunctional and/or superfluous mitochondria to maintain energy homeostasis [52]. Mitophagy pathways are stimulated by several cellular events involved in physiologic and pathophysiologic processes in different cell types, including cell differentiation, development, response to oxygen deprivation or mitochondrial damage among others [53]. In addition to the removal of damaged mitochondria, mammals can deploy specialized mitophagy pathways to remove mitochondria during different stages of development or basal conditions. Even though there are different effectors and receptors for mitophagy depending on the stimuli, a common feature is the microtubule-associated protein 1 light chain 3 (LC3) region of these proteins, which promotes mitochondrial sequestration [54]. Thus, mitophagy shares some common signaling pathways and key regulatory proteins with macroautophagy (LC3, p62 among others), there are also some specific mitochondrial proteins that either trigger or facilitate mitochondrial degradation [55].

#### 1.3.1. The PINK1/Parkin Pathway

In healthy mitochondria, PTEN-induced putative kinase 1 (PINK1) acts as a stress sensor depending on mitochondrial potential. Under normal conditions, PINK1 is rapidly imported into mitochondria and undergoes cleavage and degradation. When mitochondrial potential is dissipated, PINK1 is stabilized on the outer mitochondrial membrane and recruits Parkin, which is a cytosolic E3 ubiquitin ligase that after being phosphorylated by PINK1, activates its E3 ligase activity. Parkin ubiquitinates various outer mitochondrial membrane proteins leading to subsequent autophagosomal engulfment and lysosomal degradation [54]. Recent studies have suggested that the PINK1/Parkin pathway is involved only in stress-activated mitophagy, in a mitochondrial potential-dependent way, while basal mitophagy is independent from these proteins’ activation and subsequent mitophagy [56]. Mitochondrial clearance balance is not only affected by the PINK1/Parkin pathway, but there is also a selective-removal mitophagy depending on the different mitochondrial stress stimuli [54].

#### 1.3.2. BNIP3/NIX Pathway

BCL2 and adenovirus E1B 19-kDa-interacting protein 3 (BNIP3) and BNIP3-like (BNIP3L, also known as NIX) are mitochondria-localized proteins that are related to the BH3-only family. BNIP3 is primarily localized to the mitochondria and it is well documented that it induces loss of mitochondrial membrane potential, mitochondrial dysfunction and the activation of the mitochondrial pathways of apoptosis [57]. In addition to this role, it has been recently reported that BNIP3 induces mitochondrial autophagy in the absence of mitochondrial membrane permeabilization and Bax/Bak apoptosis proteins [57]. Moreover, Zhang et al. described an interaction between both the aforementioned mitophagy pathways, where BNIP3 interacts with PINK1 to inhibit its clearance, promoting Parkin recruitment and the initiation of PINK1/Parkin-mediated mitophagy [58].

## 2. Mitophagy and Associated Disorders in Skeletal Muscle

Skeletal muscle is characterized by its structural and functional plasticity, which provides an adaptation to variable physiological stimuli [59]. These physiological conditions are classified as acute and chronic changes related to physical activity and muscle workload. Skeletal muscle contracts due to changes in contractile machinery, Ca^2+^ concentration, and the metabolic capacity for energy production in the form of ATP that is carried out inside the mitochondria. Therefore, the mitochondrial network has evolved so that the mitochondrial production of ATP is adequate and can rapidly respond to the demands and requirements imposed by the activity in the muscle [60,61,62]. Thus, skeletal muscle mitochondria can be classified as subsarcolemal (SS) or intermyofibrillar (IMF) depending on the subcellular location. On the one hand, SS mitochondria are characterized by a large laminar shape located below the sarcolemma, while IMF mitochondria are smaller, more compact, and located among the contractile filaments [63,64,65].

Recently, three-dimensional electron microscopy studies revealed that skeletal muscle mitochondria could be classified into paravascular mitochondria (PVM), I-band mitochondria (IBM), fiber parallel mitochondria (FPM), and cross-fiber connection mitochondria (CFCM). These four types are interconnected to form a complex mitochondrial network and to be able to rapidly distribute energy through the myofibers [62]. Furthermore, skeletal muscle mitochondria serve as cellular sensors of metabolic demand and stress, where various forms of metabolic stress, including exercise, trigger responses in muscle mitochondria, generating signals corresponding to other compartments or cell tissues.

Loss or decrease in skeletal muscle function has serious consequences. In particular, the progressive decline in age-associated skeletal muscle function is related to metabolic dysfunction, susceptibility to chronic disease, loss of mobility, and an overall increase in mortality rate [66,67,68,69]. At the same time, this event is associated with the loss of mitochondrial protein homeostasis, which has been suggested to play a causal role in age-related skeletal muscle decline [70,71,72,73]. Sarcopenia is defined as a multifactorial syndrome that occurs with age and results in loss of skeletal muscle mass and its function [74]. This muscle mass loss begins to manifest approximately from 30 years of age. From then on, between 3 and 8% of muscle mass is lost, a phenomenon that accelerates significantly after the age of 60, with up to 40% of muscle mass losing after reaching 80 years of age [75,76]. This muscle mass decline has also been associated with loss in muscle function and strength, phenomena that are also related to mitochondrial dysfunction [77]. The causes of sarcopenia have been studied for many years and several hypotheses have been presented; however, the mechanisms that cause sarcopenia are still a matter of debate. Although physical changes begin to manifest at advanced ages, recent studies have shown that changes at the cellular and molecular levels precede the symptomatology of sarcopenia [78,79].

Mitochondrial biogenesis has been the focus of research during the last years and several research studies have linked this process with dysfunctional mitochondria in aging. Particularly in skeletal muscle, it has been reported that Nrf2 deficiency exacerbates frailty and sarcopenia during aging, at least partially by impairing skeletal muscle mitochondrial biogenesis and dynamics in an age-dependent manner [80,81]. Moreover, deep sequencing analysis of the mtDNA mutator mouse, a mouse model with a proofreading-deficient mtDNA polymerase γ (POLG) and a valuable model system to study the contribution of mitochondrial dysfunction to aging, showed that the increase in mitochondrial biogenesis did not reduce the accumulation of mtDNA mutations but rather caused a small increase. These results indicate that increased muscle PGC-1α expression can improve some premature aging phenotypes in the mutator mice without reverting the accumulation of mtDNA mutations [82]. Studies in humans describe a significant decrease in PGC-1α in old individuals, together with a decrease in mitochondrial dynamics regulatory proteins. Conversely an increase in TOM22 and other protein import machinery proteins has been observed [71], suggesting a possible role of import machinery and mitochondrial proteostasis in aged muscle. In this regard, some studies indicate that skeletal muscle mitochondria in elderly humans and animals show accumulation of protein damage and release of ROS [70,83] that would contribute to global mitochondrial compromise and thus, to dysfunctional skeletal muscle tissue [83,84]. Studies in aged skeletal muscle in mice and humans have demonstrated altered expression of apoptotic and antiapoptotic proteins and enhanced susceptibility towards mitochondrial transition pore opening [85]. This recent evidence shows that the relationship between sarcopenia and mitochondria is still a matter of debate when defining the molecular mechanisms of muscle mass and function loss during aging.

**Figure 2:**
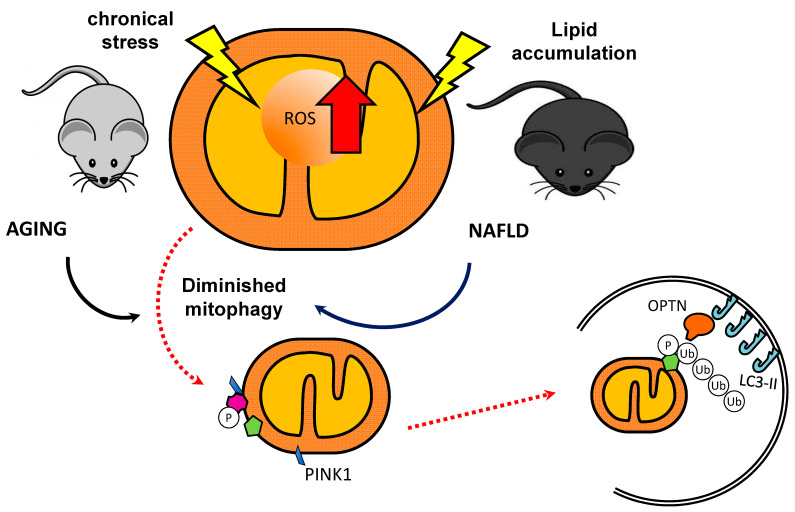
Diminished mitophagy in NAFLD and Sarcopenia. Mitophagy-associated proteins are decreased during the aging process and have been linked with aging associated diseases. The accumulation of damaged mitochondria because of impaired mitophagy could be involved in the development of disease and organ malfunction.

Concerning mitophagy in skeletal muscle, current studies have described the decrease in this process with aging. In aged-mice skeletal muscle, PINK1 expression was reduced when compared to young mice muscle [86]. Interestingly, in a study with humans, despite no difference in Parkin expression between young and older muscles being observed, a lower ratio of Parkin relative to voltage-dependent anion channel (VDAC) in aged muscle was found, suggesting that the mitophagic potential may be impaired with aging (Figure 2) [84].

Although there are few reports about muscle-specific silencing of autophagy/mitophagy genes, the most studied is the deletion of the autophagy related protein 7 (Atg7) gene, which encodes for a crucial component of both, Atg12 and LC3 ubiquitin-like conjugation systems involved in the expansion of the autophagosome membrane [87]. The deletion of the Atg7 gene, and subsequent defective mitophagy, causes an augmentation in protein carbonylation and accumulation of enlarged, swollen mitochondria in skeletal muscle and significantly shortens lifespan [88]. Thus, the decrease in mitophagy in skeletal muscle would be analogous to the characteristic mitochondrial phenotype traits related to aging [17]. Additionally, a higher accumulation of the autophagy markers p62 and LC3-II was observed in skeletal muscle of aged Fisher 344 BN rats [89], which may indicate diminished autophagic degradation in aging muscle, because LC3-II is catabolized during mitophagy. Furthermore, AMP-activated protein kinase (AMPK) plays a vital role in autophagy and mitophagy during aging and its activation in skeletal muscle seems to be diminished during senescence [90]. In response to muscle specific AMPK deletion, both SS and IMF mitochondrial sizes increased, and a significant reduction in mtDNA was observed [91]. Defects in mitochondrial metabolism provoke a decline in tissues and/or organs function. Likewise, malfunctioning of the mitochondrial proteostasis mechanisms owing to insufficient or impaired mitophagy may have an important effect upon homeostasis throughout the body. Mutations in Bcl2 phosphorylation sites inhibit the release of Beclin1 from the Bcl2-Beclin1 complex preventing the activation of autophagy, impairing exercise-induced increase in insulin sensitivity and failing to increase the translocation of GLUT-4 to the plasma membrane in skeletal muscle [92]. Moreover, it has been demonstrated that the inhibition of autophagy produces decreased endurance, altered glucose metabolism during exercise and impaired exercise-mediated protection against glucose intolerance induced by high fat diet [92].

Physical activity and muscle contraction have been established as powerful factors regulating mitophagy, which may potentially correct or attenuate dysfunctional mitophagy, during metabolic stress [93] and nutritional deficits such as cachexia [94]. In C26 tumor implanted mice, exercise attenuated cachexia-induced p62 and LC3 II/I accumulation indicating improved mitophagy. Additionally, AMPK activation via 5-Aminoimidazole-4-carboxamide ribonucleotide (AICAR) administration suppressed p62 accumulation through promotion of mitophagy and accelerating the turnover of p62 accumulation in cachectic muscle [94]. Therefore, exercise and/or muscle contraction might be an effective therapy for the restoration of mitophagy balance, which would lead to proper mitochondrial and metabolic homeostasis of skeletal muscle. Thus, multiple studies point out the role of mitophagy over skeletal muscle under different metabolic conditions. However, additional investigations are required to establish a direct mechanism in the regulation of these processes. To deepen on the aforementioned view, several studies have attempted to understand the effects of resistance training on cellular systems over muscle aging. While some conflicting findings have been observed, it is almost undeniable that acute aerobic exercise is likely to increase autophagic responses in skeletal muscle [95]. For instance, it has been observed that aging muscles show higher expression of autophagic markers such as ATG7 and Beclin-1 when they undergo endurance training. Both markers are important in the autophagosome formation [96]. Furthermore, physically active elderly subjects have increased mRNA levels of autophagy markers such as Beclin-1, ATG7 and p62. Likewise, they also have increased mitophagy markers including BNIP3 and Parkin in skeletal muscle [97]. Therefore, it is possible that endurance training or chronic muscular activity might lead to a remodeling of the mitochondrial network in the skeletal muscle of elderly individuals, although, further studies are needed to fully understand these observations [96].

The decline of skeletal muscle oxidative capacity is a common feature of chronic diseases such as type 2 diabetes (T2D) [98], chronic obstructive pulmonary disease (COPD) [99] and congestive heart failure (CHF) [100]. Functional deficiencies, such as muscle dysfunction and decreased exercise capacity, are associated with lower muscle oxidative capacity that could eventually lead to disabilities or even handicaps. Additionally, impaired muscle oxidative capacity has been suggested to accelerate muscle wasting [101] and may increase the metabolic and cardiovascular risks [102]. Certainly, skeletal muscle mitochondrial quantity and its oxidative capacity are affected in chronic diseases. Although the number of studies on mitophagy in skeletal muscle of patients with the aforementioned chronic diseases is small, an increased presence of proteins related to mitophagy, such as BNIP3 and PARK2, has been observed together with a positive regulation of autophagy in vastus lateralis muscle of patients with COPD [103]. On the contrary, Kruse et al. found no differences in markers related to autophagy/mitophagy in vastus lateralis muscle of type 2 diabetes subjects [104]. However, none of these investigations evaluated mitochondrial content or oxidative capacity.

In skeletal muscle, Sakellariou et al. provided evidence that the regulation of mitochondrial ROS production could be affecting the PINK1/Parkin mitophagy pathway, without having any incidence in muscle atrophy [105]. In addition, recent evidence has shown that mitophagy mechanisms in high metabolic demand tissues, such as skeletal muscle, are independent of PINK1 activation in basal conditions [56]. On the other hand, Gouspillou et al. described that ablation of Parkin in skeletal muscle increases basal autophagic flux and BNIP3 levels, whereas skeletal muscle fibers from Park2 KO mice show increased mitochondrial dysfunction, together with a higher cross-sectional area of muscle fibers [106]. Following this research line, Peker et al. reported that Parkin KO muscles had dysfunctional mitochondria, but, in controversy with Gouspillou et al., Parkin KO muscles exhibited smaller myofiber area [107]. Other studies have reported that aging-induced Mitofusin-2 deficiency triggers a ROS-dependent adaptive signaling pathway through induction of HIF1α transcription factor and BNIP3 [108]. Studies in humans have described decreased mitophagy-related genes in skeletal muscle of physically inactive elderly women and a positive correlation between this decrease and the physical function or leg lean mass [95].

Since mitochondrial fission is usually linked to the mitophagy process, it could be considered as an indirect indicator of mitophagy. Thus, the decreased average mitochondrial size observed in the vastus lateralis muscle of patients with CHF [109] or T2D [110], suggests a mitochondrial fission/fusion balance inclined towards mitochondrial fission. Moreover, decreased expression of Mfn-2 is observed in skeletal muscle of type 2 diabetes patients [111], with no statistically significant difference being reported in skeletal muscle of CHF patients [112]. In spite of the unchanged gene expression, Molina et al. demonstrated that the protein expression of Mfn-2 decreased in skeletal muscle of CHF patients compared with sedentary subjects [113]. In relation to these mitochondrial variations in atrophy and aging, Romanello et al. described that the induction of mitochondrial fission by overexpressing proteins of the fission machinery produces a decrease in the area of muscle fibers and an increase in autophagy [114]. Along with these results, other studies have described a decrease in proteins linked to mitochondrial dynamics as age increases, thus relating mitochondrial morphology to aging [115]. The deficiency of mitochondrial fission processes during aging, could promote mitochondrial dysfunction due to the accumulation of damaged mitochondria, considering that mitochondrial fission processes precede mitophagy and maintain the homeostasis of these organelles [116]. In the context of studying mitochondrial morphology in aging, Leduc-Gaudet et al. reported highly fused mitochondrial structure in aged mouse muscle [117].

Nevertheless, as discussed above, the modifications in mitochondrial morphology during aging are still a matter of debate and none of these studies have shown how the proposed mechanisms are altered in middle age. Only a few recent studies have focused on describing changes prior to the state of manifesting severe sarcopenia symptoms. In this regard, del Campo et al. reported time-line changes in mitochondrial morphology and distribution in adult skeletal muscle fibers [79]. The results show that a mitochondrial network fused-like phenotype can be observed in 10–14-month-old mice. Moreover, muscle function and cross-sectional area begin to decline at the same age [79]. In addition to those findings, Sayed et al. showed that mice at an early stage of aging already display significant changes in muscle mitochondrial morphology and ultrastructure, which are suggestive of the onset of sarcopenia. Their results describe a significant decrease in the muscle-fiber cross-sectional area and an increase in intermyofibrillar mitochondria in skeletal muscle [78]. Altogether, these findings suggest that during middle age an impairment of mitochondrial homeostasis might occur previously to the development of sarcopenia symptoms and, consequently, this turnover point could be an interesting target to intervene. Likewise, it would be important to determine whether other mitochondrial mechanisms could be influencing the development of sarcopenia across the aging process, to shed light about imbalanced mitophagy in the course of chronic diseases and aging.

Given the aforementioned, the question is whether mitophagy in skeletal muscle would be a beneficial or harmful process. Mitophagy is vital for mitochondrial quality control and therefore a diminution or an alteration in this process could result in extensive mitochondrial damage, mitochondrial dysfunction, or cell death [118]. In this context, it would be more adequate considering mitophagy as a cellular process only, not intrinsically good or bad, but inclined toward one or the other outcomes according to the cellular needs at any given time.

In this respect, it seems logical relating mitophagy and UPR^mt^. The first process eliminates damaged components or eventually the whole organelle; the second one triggers the expression of proteases and chaperones to relieve mitochondrial stress provoked by misfolded proteins and promotes biogenesis to maintain proteostasis and mitochondrial function [119]. Therefore, both events could be connected through a common stressor mechanism that may benefit mitochondrial populations suitable for recovery in detriment of those tagged for mitophagy. While mitophagy tends to enclose the damage of dysfunctional mitochondria, stress responses such as UPR^mt^ facilitate the recovery of mitochondria susceptible of being saved, resulting in a healthier mitochondrial network [35].

## 3. UPR^mt^ in Sarcopenia

Age-associated atrophy contributes to decreased mobility and increased fragility, where many of the changes underlying the adaptive and plastic nature of the muscle converge into mitochondria. Thus, the maintenance of a functional mitochondrial network is critical for the preservation of skeletal muscle throughout life. During aging a significant increase in defective mitochondria is produced, which drives to a decline in the overall physiological functioning of the organism. This mitochondrial dysfunction is accompanied by reduced oxygen consumption with a corresponding diminution in respiratory complex activity [120]. Interestingly, this decline has been linked to the development of many age-related diseases such as Parkinson’s and coronary artery disease [121]. In a metabolic context, Nargund et al. found that the UPR^mt^ activation was a positive regulator of glycolysis’ genes transcription suggesting that this compensatory mechanism promotes an alternative form of ATP production [122]. Concomitantly, OXPHOS transcript accumulation is diminished to match OXPHOS complex biogenesis rates to the protein folding and complex assembly capacity of the dysfunctional organelles, to finally restore mitochondrial respiration in an efficient manner [123].

Considering that skeletal muscle is one of the main metabolic regulators and the importance of mitochondrial homeostasis in its function, it is possible to establish a correlation between mitochondrial dysfunction and the decrease in muscle mass, where the UPR^mt^ would have a fundamental role. In this regard, Chung et al. found that muscle-specific Crif1 (CR6-interacting factor 1) knock-out mice activated the UPR^mt^, stimulated the production of mitokines that regulate systemic energy homeostasis and induced progressive mitochondrial OXPHOS dysfunction, ultimately producing advanced muscular dystrophy and sarcopenia. In addition, it was observed that accumulation of abnormal, swollen mitochondria with disrupted cristae and some mice manifested traits of mitochondrial myopathy reduced muscle mass and lowered strength [124]. Tamura et al. stated that mitochondrial and endoplasmic reticulum stress and the activation of their compensatory mechanisms (UPR^mt^ and UPR^er^) are involved in the pathogenesis of sarcopenia. Interestingly, heat shock treatment has been suggested as a strategy to counteract sarcopenia, improving age-induced mitochondrial and ER stress. In this regard, heat stress showed notable improvements in age-related changes in soleus muscle, but minor effects in gastrocnemius and plantaris muscles, suggesting that the outcomes vary qualitatively according to the skeletal muscle type [22]. Although these findings seem promising, more studies are needed to strengthen the concept of heat stress as a potential treatment for sarcopenia.

One of the first described physiological functions of UPR^mt^ is associated with the lifespan extension observed after mild perturbations of OXPHOS in *C. elegans* [125]. The mitochondrial disturbances that lead to the lifespan extension also cause activation of the UPR^mt^ [126] and it is important to note that the increased longevity of the *C. elegans* model requires multiple components of the UPR^mt^, such as histone lysine demethylases jmjd-3.1, matrix peptide exporter haf-1 and chaperone dve-1, which demonstrates that the pathway is protective during mitochondrial dysfunction [127]. However, how the UPR^mt^ contributes to extending longevity remains unclear. In this context, the mitochondrial homeostasis and its variations are associated to lifespan and longevity, considering that mitochondrial dysfunction is frequently a characteristic of aging and different diseases. Many investigations have shown a positive correlation between UPR^mt^ and longevity in *C. elegans*, Drosophila and mice [126]. However, this relationship is not straightforward because the UPR^mt^ activation might lose its beneficial status and become detrimental, if the UPR^mt^ activation is chronic [128], since the long-term implications of a constitutively active UPR^mt^ promotes glycolytic and biosynthetic processes, which are characteristic traits of highly proliferative cells [35].

As organisms possess a high number of mtDNA in each cell, a small amount of mutated mtDNA is well tolerated possibly due to a much higher percentage of healthy mtDNA. However, deleterious mtDNA can accumulate as the organism grows old and disturb OXPHOS, impairing mitochondrial homeostasis and ultimately cellular function [129]. For instance, in a model of deleterious heteroplasmy in *C. elegans*, the UPR^mt^ is activated, since the mtDNA lacks the genes necessary for the expression of various OXPHOS subunits [128]. The activation of UPR^mt^ likely occurs in an attempt to maintain proteostasis and promote recovery of mitochondrial dysfunction [128]. Interestingly, the elimination of ATFS-1 resulted in a preferential reduction in mutated mtDNA together with an increase in wild-type mtDNA. In addition, the constitutive activation of the UPR^mt^ in heteroplasmic worms was sufficient to cause an increase in mitochondrial biogenesis, which resulted in a preferential increase in deleterious mtDNA over the wild-type [128,130]. These data demonstrate that the activation of the UPR^mt^ results in the spread of harmful mtDNA, although the mechanism is unclear. Similar results were observed in a mouse model of mitochondrial myopathy caused by an aberrant accumulation of mutated mtDNA, resulting in muscle OXPHOS impairment [131,132]. Interestingly, the inhibition of mTORC1, a factor that induces UPR^mt^, resulted in a reduction in the activation of UPR^mt^ and limited the accumulation of deleterious mtDNA, which, in turn, reduced the progression of mitochondrial myopathy [131]. Furthermore, this inhibition limits mitochondrial biogenesis and induces an increase in mitophagy, suggesting a mechanism by which the UPR^mt^ could antagonize mitophagy and promote the accumulation of harmful mtDNA [133].

In relation to exercise and stress response, a single bout of resistance exercise provokes the activation of the UPR, similar to what is observed after endurance training [134]. During exercise, both the UPR^er^ and the UPR^mt^ are upregulated during early stages of training. In addition, several investigations have studied the expression level of UPR genes and have shown that those gene variations occur according to the type of exercise training program [135,136,137]. The UPR activation precedes changes in mitochondrial biogenesis and autophagy, which together promote mitochondrial adaptations [23]. These data would suggest that the activation of the UPR might mediate some of the exercise-induced changes observed in skeletal muscle, but more evidence is required [23,134,138]. In this respect, it has been stated that physical activity improves metabolism and physical performance of old mice. Aerobic exercise (i) increases important proteins involved in mitochondrial functions and biogenesis, such as VDAC and SIRT1, along with mitochondrial encoded genes in skeletal muscle of aged mice; (ii) induces a mitonuclear imbalance, increasing the MTCO1/ATP5α ratio and UPR^mt^ markers in skeletal muscle, including levels of protein Hsp60, LONP1 and Yme1L1 in gastrocnemius muscles of elderly mice [21]. Thus, these data suggest that the UPR^mt^ activation could be triggered at least in part by exercise, improving mitochondrial metabolism and oxidative capacity in elderly individuals. Some studies support the idea that increasing the stress signaling via the UPR^mt^ through the modulation of NAD^+^ levels may be a target to ameliorate mitochondrial function in aged tissues [139]. Supporting this idea, it has been observed that treatment with the NAD^+^ precursor nicotinamide riboside (NR) induces the UPR^mt^, which has an effect on mitochondrial activity modulating muscle stem cells senescence [140]. Considering the aforementioned, it would be reasonable to target mitochondria in search for treatment of sarcopenia. In this regard, resveratrol and polyphenols that enhance nuclear/mitochondrial protein interactions have not yet shown major effects in skeletal muscle [141,142]. Additionally, FGF21 has been shown to be a direct ATF4 target downstream of the UPR^mt^ [143], while GDF15 secretion is induced in a CHOP-dependent manner during UPR^mt^. In several mouse models of defective mitochondria, skeletal muscle secretes FGF21 into circulation to exert an endocrine effect [143]. It has also been seen that GDF15 is induced and released from skeletal muscle upon multiple forms of mitochondrial stress [14]. The participation of these myokines in the signaling of UPR^mt^ and mitochondrial maintenance may provide a partial scenario of how the organism communicates and manifests similar age-related mitochondrial adaptations in different organs (Figure 1), despite their differences in mitochondrial morphology due to cellular activity and energy needs [4].

## 4. Mitophagy and Non-Alcoholic Fatty Liver Disease (NAFLD)

As previously shown, mitophagy is imperative for the maintenance of the mitochondrial network, which is probably impaired in the process of aging, at least in skeletal muscle. Despite liver and skeletal muscle possess different energy demands and mitochondrial morphology, several studies have demonstrated that the efficiency of mitophagy is impaired during the aging process [144] and in the context of liver disease associated with aging or unhealthy eating habits (Figure 2) [145]. The evidence is clearer when it comes to possible therapeutic targets and pharmacological compounds that could address the pathophysiology of NAFLD through the modulation of mitophagy processes [146]. In this respect, an in vitro study proposes that liraglutide, a long-acting GLP-1 analog, attenuates mitochondrial dysfunction and ROS generation, with concomitant PINK1-mediated mitophagy enhancement and protection against lipid accumulation [147]. Besides, the anthocyanin cation cyanindin-3-*O*-glucoside improves NAFLD by promoting PINK1-mediated mitophagy in in vitro and in vivo models [148]. On the other hand, Li et al. demonstrated that high fat diet-induced liver damage is associated with Sirt3 downregulation linked to BNIP3-mediated inhibition of mitophagy, causing hepatocytes to undergo mitochondria-dependent death pathways [149]. These data point to the regulation of BNIP3 as a promising therapeutic target [150].

## 5. UPR^mt^ and NAFLD

Development and progression of NAFLD are associated with a high energy intake and consumption of specific nutrients such as saturated fatty acids (FAs) (i.e., palmitate) [151], trans FAs [152] and carbohydrates (i.e., glucose, fructose) [153]. Besides, derangement in the intake of n-6 long-chain polyunsaturated FAs (n-6 LCPUFAs) and n-3 LCPUFAs enhancing the n-6/n-3 LCPUFA ratio [154] also occur, promoting the abnormal deposition of triglycerides (TGs) in the liver. The pathogenic mechanisms underlying NAFLD are beginning to be understood, placing the development of a drastic and progressive oxidative stress as a crucial molecular phenomenon triggering hepatocellular injury [155]. This redox imbalance is related to an enhanced and sustained ROS production from the start-point of overnutrition until NAFLD development, in which the initial excess FAs can be rapidly oxidized with consequent superoxide radical (O_2_^•−^) and hydrogen peroxide (H_2_O_2_) generation at complex I of the mitochondrial respiratory chain [156,157]. These processes are facilitated by the development of insulin resistance (IR) [158], which underlies the production of a pro-inflammatory state with upregulation of tumor necrosis factor-alpha (TNF-α) generation, due to activation of Kupffer cells [159], liver macrophages that contribute to ROS production through NADPH oxidase operation [160]. Under these conditions, the expression of liver microsomal cytochrome P450 2E1 is drastically enhanced [161,162], an enzyme characterized by its large capacity for ROS generation [163]. Consequently, liver oxidative stress in NAFLD leads to the oxidative deterioration of biomolecules, including PUFA peroxidation, DNA oxidation [164] and protein carbonylation. The latter alteration is observed in experimental (high-fat diet (HFD) with 60% of fat for 12 weeks) [165] and human [155] obesity, constituting a proteotoxic stimulus that may trigger UPR^er^, UPR^mt^ or both.

Prolonged liver UPR^er^ is associated with enhancements in the cellular levels of oxidized, unfolded proteins and saturated FAs such as palmitate, producing selective, structural effects in the ER. In the case of palmitate, this FA is incorporated into either TGs, forming the insoluble tripalmitin that is retained in the ER, or into phospholipids, disrupting the ER architecture [166]. Under these conditions, two branches of the UPR^er^ are activated, namely, (i) the PERK-eIF2α-ATF4 pathway promoting de novo FA synthesis and TG formation via upregulation of the transcriptional regulator SREBP-1c; (ii) the IRE1α-XBP1 branch triggering TG synthesis via expression of PPARγ and the C/EBPs [167], thus inducing a steatotic response. Since NAFLD is a progressive disorder, chronic UPR^er^ was proposed to favor the development of more drastic forms of the disease, including inflammatory and fibrotic responses ensuing liver failure [167,168].

Liver mitochondria are structurally and functionally deranged in NAFLD and its progressive form of injury non-alcoholic steatohepatitis (NASH) [169], features that can result from the interruption of the electron flow through the mitochondrial respiratory chain (MRC) that favors the direct transfer of electrons to O_2_ to produce O_2_^•−^ and H_2_O_2_ [170]. Under conditions of ROS overproduction at the mitochondrial level, several alterations are induced, namely, (i) the oxidation of cardiolipin that destabilizes MRC complexes [171]; (ii) the stimulation of lipid peroxidation processes producing malondialdehyde and 4-hydroxynonenal, LCPUFA oxidation products that are inhibitors of cytochrome-c oxidase [170]; (iii) the direct oxidative deterioration of MRC complexes and mtDNA [170,171]. mtDNA oxidation is related to a lack of protective histones and limited repair mechanisms, thus making mtDNA highly susceptible to ROS, compromising the expression of components of the electron flow and ATP synthesis processes [170,172]. Consequently, mitochondrial dysfunction ensues in HFD-induced NAFLD, which is characterized by low activities of citrate synthase, MRC complex I and II [173], succinate dehydrogenase and cytochrome-c oxidase [174], concomitantly with a fall the respiratory control ratio [174], the NAD^+^/NADH ratio and the ATP content [173]. Importantly, age increases the susceptibility to HFD (48% of fat for 9 weeks)-induced hepatic steatosis, which is related to lower mitochondrial mass and FA oxidation and higher ROS generation [175]. Besides mitochondrial dysfunction in HFD-induced NAFLD [173,174], derangement of mitochondrial biogenesis is also encountered [169]. Mechanistically, this effect of HFD is associated with downregulation of hepatic PPAR-α-FGF21-AMPK-PGC-1α signaling cascade [173], in which loss in the activity of the transcriptional activator PGC-1α is related to (i) diminished PGC-1α mRNA expression; (ii) lower AMPK expression compromising PGC-1α activation by phosphorylation [176]; (iii) decreased NAD^+^-dependent Sirtuin 1 (SIRT1) activity by reduction in the NAD^+^/NADH ratio lowering PGC-1α activation by deacetylation [177]; (iv) failure in the physical interaction between PGC-1α and NRF-1 to promote mitochondrial biogenesis [178].

In general terms, the UPR^mt^ is associated with several pathologies sharing a dysfunctional state of mitochondria induced by a proteotoxic stress [14], such as in NAFLD [173,174]. The response is understood in terms of a retrograde signaling pathway establishing a mitochondrion-to-nucleus communication to enhance the expression of chaperones and proteases as an adaptive mitohormetic mechanism [169,170]. Although UPR^mt^ is considered to be useful to restore or prevent metabolic alterations leading to the development of NAFLD [161,169], the subject has received little attention at preclinical and clinical levels. In this context, studies using an alternate NAFLD model to the standard HFD (60% as fat for 12 weeks), namely, a high-fat (44.6%) high-sucrose (40.6%) (HFHS) diet for 18 weeks, revealed the development of steatosis and mitochondrial dysfunction with NAD^+^ and ATP depletion, concomitantly with enhanced mRNA expression of lipogenic, inflammatory and fibrotic markers [25]. Under these conditions, however, the HFHS diet did not alter the mRNA levels of the UPR^mt^ markers ClpP, Hsp10 and Hsp60, despite the enhancement in those of UPR^er^ [25]. Interestingly, HFHS diet-induced chronic hepatosteatosis was prevented or reversed by inducing UPR^mt^ by means of supplementation with the NAD^+^ precursor nicotinamide riboside, an effect that was suggested to involve SIRT1, SIRT3 upregulation (Figure 1) [25]. The aforementioned allows one to establish the induction of the UPR^mt^ by NAD^+^ replenishment as a potential common denominator treatment for diverse myopathies and NAFLD. However, it is yet to be determined if these improvements are owing to the UPR^mt^ activation. These data point to abrogation of mitochondrial dysfunction as a crucial mechanism underlying NAFLD prevention, in agreement with a recent report using the co-administration of docosahexaenoic acid (DHA) and hydroxytyrosol (HT) along with HFD feeding [173], which warrants the assessment of UPR^mt^ markers. Furthermore, the above strategy may also be important in the treatment of the more severe condition of NASH, since NASH patients subjected to a mixture of n-3 LCPUFAS (α-linolenic acid (ALA), EPA and DHA) for 6 months exhibit improvement of mitochondrial function with induction of the chaperone HSP60, in addition to attenuation of hepatic lipogenesis and ER stress [179].

In the scenario of obesity-induced NAFLD, the liver develops not only UPR^er^ and mitochondrial dysfunction [170] but also physical communications between ER and mitochondria are established, defined as mitochondrial associated ER membranes (MAMs) [180], which are crucial in determining cell viability during nutrient overload. These MAMs are structural bridges that primarily exchange Ca^2+^, but also lipids and ROS, for which a structural and functional integrity is required to manifest normal ER-mitochondrial communications [181]. After disruption of MAM integrity, Ca^2+^ homeostasis is lost in association with UPR^er^, mitochondrial dysfunction, oxidative stress and IR, factors involved in NAFLD development and progression [181]. In this respect, genetic (ob/ob mice) and dietary (HFD) models of obesity showed reorganization of MAMs in the liver, with increased MAM formation resulting in higher Ca^2+^ flux from ER to mitochondria, enhanced oxidative stress and mitochondrial metabolic alterations [182]. Specifically, obesity upregulated the expression of proteins related to ER to mitochondria Ca^2+^ flux, namely, the Ca^2+^ channels IP3R1 and IP3R2, concomitantly with that of the ER-mitochondrial tethering protein PACS-2 [182]. Although normal links are necessary for adequate propagation of Ca^2+^ signals from ER to mitochondria, excessive MAM formation is deleterious to cells as in NAFLD, supporting the dependence of cell function and survival on the proper spacing between ER and mitochondria [183]. In this context, adenovirus-mediated knockdown of liver IP3R1 and PACS-2 improves mitochondrial function and metabolic performance pointing to MAM formation as a therapeutic target for NAFLD [182]. This could be approached with protocols abrogating mitochondrial dysfunction such as nicotinamide riboside [25] or DHA and HT co-administration [173] on future studies.

## 6. Conclusions

A thoughtful understanding of the cellular mechanisms underlying the physiological processes involved in sarcopenia and NAFLD is fundamental in order to develop therapeutic tools to attack the actual cause of the dysfunction. Despite this review being centered on the two aforementioned pathologies, the UPR^mt^ seems to be related to many more, which is consistent with mitochondrial dysfunction as a common feature for diverse diseases. In fact, mitochondrial malfunctioning is present in various age-related pathologies. To unveil the precise mechanisms implicated would permit one to distinguish the failing step of the pathway, which would have profound clinical implications, because it might allow one to establish potential therapeutic targets. For example, increased levels of transcripts associated with UPR^mt^ activation have been observed in patients with myopathy caused by mitochondrial disorders [184]. This observation is consistent with the activation of the UPR^mt^, but to be used as a therapeutic advantage is necessary to determine the mitochondrial-stress-induced transcription factors elicited in each pathological scenario, together with the effects of the transcriptional response over physiology.

Altogether, the above research suggests a major role of mitochondrial adaptations and removal in the pathogenesis of metabolic diseases (Table 1), nevertheless the results are still controversial, and the definitive mechanisms are far from being unveiled. In this context, sarcopenia and NAFLD exhibit pronounced mitochondrial dysfunction, thus compromising cell viability due to energy collapse. Mitochondrial dysfunction is associated with (i) mutations in genes encoding OXPHOS proteins and ATP synthase; (ii) oxidative stress due to excessive ROS generation that triggers the accumulation of damaged mtDNA, protein carbonylation and unfolding and phospholipid peroxidation particularly that of cardiolipin that destabilizes MRC complexes. Under these conditions, the canonical UPR^mt^ axis is activated to promote cell survival and the recovery of the mitochondrial network to ensure optimal cellular functions. UPR^mt^ activation underlying oxidative stress and ER stress conditions involves the phosphorylation of eIF2α by kinases GCN2 and PERK, phosphorylated eIF2α being able to attenuate global translation while favoring that of CHOP, ATF4 and ATF5. The latter transcription factors induce the expression of chaperones, proteases, antioxidant components limiting ROS toxicity, OXPHOS complex assembly factors and protein import components, which afford the recovery of mitochondrial functioning [5,12,20] (Figure 1). Besides UPR^mt^ activation, mitophagy upregulation is also established to eliminate dysfunctional or altered mitochondria via PINK1/Parkin and BNIP3/NIX pathways. Although UPR^mt^ activation and mitophagy provide protection under conditions of mild mitochondrial dysfunction underlying sarcopenia and NAFLD, future time-course studies are required to assess the effectiveness of strategies contributing to the recovery of proteostasis, since prolonged or dysregulated UPR^mt^ activation is deleterious to the cell [5,12,23].

## Figures and Tables

**Table 1:** UPR^mt^ in the context of skeletal muscle and liver.

Reference	Model	Main Findings
Cordeiro et al., 2020 [21]	Young and old mice underwent 4 weeks of aerobic exercise training	Increased UPR^mt^ markers in gastrocnemius muscle of aged mice
Tamura et al., 2017 [22]	Young and aged mice received heat stress treatment	Remarkable improvements in age-related changes in soleus, but minor effects in gastrocnemius and plantaris
Memme et al., 2016 [23]	Rats subjected to chronic contractile activity (CCA) for 1 to 7 days	UPR^mt^-specific markers were induced 10 to 80% between days 1 and 7
Al-Furoukh et al., 2015 [24]	ClpX overexpression in C2C12 mouse myoblasts and HEK293T cells	Upregulation of markers of the UPR^mt^
Gariani et al., 2016 [25]	Mice fed with HFHS diet plus nicotinamide riboside	Induction of a SIRT1- and SIRT3-dependent UPR^mt^, preventing/reverting NAFLD
Quirós et al., 2017 [26]	Multiomics approach in mammalian cells treated with 4 mitochondrial stressors	Identification of ATF4 as the main regulator of the stress response
Fiorese et al., 2016 [27]	*C. elegans* lacking ATFS-1 and expressing a reporter of UPR^mt^	Rescue of UPR^mt^ activation under stress conditions by ATF5
Bennett et al., 2014 [28]	Genome-wide RNAi screen for negative regulators of the UPR^mt^	UPR^mt^ is neither necessary nor sufficient for lifespan extension
Michel et al., 2015 [29]	Impairment of mtDNA expression	Triggering of ISR, but not of UPR^mt^
Zhao et al., 2002 [30]	Mutated OTC to provoke mitochondrial protein accumulation	Induction of nuclear genes encoding for Hsp60, Hsp10 and ClpP

## Abbreviations

AICAR	5-Aminoimidazole-4-carboxamide ribonucleotide
AMPK	AMP-activated protein kinase
Atg7	Autophagy related protein 7
Atg12	Autophagy related protein 12
ATP5α	ATP synthase subunit alpha
ATF-4	Activating transcription factor 4
ATF-5	Activating transcription factor 5
ATFS-1	Activating transcription factor associated with stress
BAK	BCL2 homologous antagonist/killer
BAX	BCL2-like protein 4
BNIP3	BCL2 and adenovirus E1B 19-kDa-interacting protein 3
BNIP3L	BNIP3-like
bZIP	Basic leucine zipper
C26	Colon-26 carcinoma
CEBPβ	CCAAT/enhancer binding protein β
CFCM	Cross-fiber connection mitochondria
CHF	Congestive heart failure
CHOP	CEBP Homologous Protein
ClpP	Caseinolytic protease proteolytic subunit
ClpXP	ATP-dependent Clp protease ATP-binding subunit ClpX-like
COPD	Chronic obstructive pulmonary disease
Crif1	CR6-interacting factor 1
DHA	Docosahexaenoic acid
DNA	Deoxyribonucleic acid
Dve-1	Homeobox domain-containing protein
eIF2α	Eukaryotic translation initiation factor 2 alpha
FA	Fatty acid
FGF21	Fibroblast growth factor 21
FPM	Fiber parallel mitochondria
GCN2	General control non-derepressible 2 kinase
GDF15	Growth differentiation factor 15
Haf-1	Matrix peptide exporter
HFD	High-fat diet
HFHS	High-fat and high-sucrose
HRI	Heme-regulated inhibitor kinase
Hsp10	Heat shock protein 10
Hsp60	Heat shock protein 60
Hsp90	Heat shock protein 90
HT	Hydroxytyrosol
IMM	Inner mitochondrial membrane
IMS	Intermembrane space
IP3R1	Inositol 1,4,5-triphosphate receptor type 1
IP3R2	Inositol 1,4,5-triphosphate receptor type 2
IRE1	Inositol-requiring enzyme-1
ISR	Integrated stress response
Jmjd-3.1	Lysine-specific demethylase (JuMonJi Domain protein)
LC3	Microtubule-associated protein 1 light chain 3
LONP1	Lon protease 1
MAM	Mitochondrial associated ER membrane
Mfn-2	Mitofusin 2
MPP	Mitochondrial processing peptidase
MRC	Mitochondrial respiratory chain
MTCO1	Mitochondrially encoded cytochrome c oxidase 1
mTORC1	Mammalian target of rapamycin complex 1
mtHsp70	Mitochondrial heat shock protein 70
MTS	Mitochondrial targeting sequence
LCPUFA	Long-chain polyunsaturated FA
NAFLD	Non-alcoholic fatty liver disease
NASH	Non-alcoholic steatohepatitis
NRF-1	Nuclear respiratory factor 1
OMM	Outer mitochondrial membrane
OPTN	Optineurin protein
p62	Classical receptor of autophagy
PACS-2	Phosphofurin acidic cluster sorting protein 2
PERK	Protein kinase RNA-like ER kinase
PGC-1α	Peroxisome proliferator-activated receptor gamma coactivator 1-alpha
PINK1	PTEN-induced putative kinase 1
PKR	Protein kinase RNA-activated
PPARγ	Peroxisome proliferator-activated receptor gamma
PUFA	Polyunsaturated fatty acid
SIRT1	Sirtuin 1
SIRT3	Sirtuin 3
SREBP-1c	Sterol regulatory element-binding protein-1c
TIM	Translocase of the inner membrane
TNF-α	Tumor necrosis factor-alpha
TOM	Translocase of the outer membrane
VDAC	Voltage-dependent anion channel
XBP1	X-box-binding protein-1
Yme1L1	Yme1-like 1 ATPase
